# Children with Additional Support Needs Risk Missing Out on Effective Vision Screening: Audit and Survey Considering Attendance Rates and Parent Reported Barriers to Service Access, including Recommendations for Improvement

**DOI:** 10.22599/bioj.451

**Published:** 2025-04-03

**Authors:** Cirta Tooth, Julius Rogowski

**Affiliations:** 1 Princess Alexandra Eye Pavilion, UK; 2 University of Edinburgh, UK

**Keywords:** vision screening, attendance, children, additional support needs, inequality, follow up, service access, optometrist, orthoptist, preschool, refractive error, amblyopia

## Abstract

**Objective:**

Vision screening programs in children aged 4–5 years aim to address visual issues before they start school, supporting children’s educational, developmental, emotional, and social well-being. This study evaluates attendance rates and barriers to attendance for children requiring follow-up in an urban hospital eye service after their initial screening visit.

**Methods and Analysis:**

Retrospective data on attendance, visual acuity, refractive errors, and presence of additional support needs (ASN) were collected from the National Database for preschool screening and the hospital electronic record system. Caregivers of children with missed appointments were invited for a telephone survey.

**Results:**

First-time hospital attendance rate was 61%. Children with ASN were 1.8× more likely to miss two hospital appointments and had more incomplete tests compared to children without ASN. In children receiving a spectacle prescription, vision improved by 0.07 LogMAR in the better eye and 0.16 LogMAR in the worse eye. Barriers for attendance included being unaware of the appointment, ASN, hospital environment, scheduling and transport issues. Parents recommended information with the appointment letter in an accessible language for the child and caregiver, an appointment reminder text message and options for vision tests in the community or at school.

**Conclusion:**

Whilst the initial uptake of vision screening is high, there is a significant proportion of children with incomplete screening tests or missed follow-up appointments. Greater attention should be placed on improving accessibility of the service for children with ASN, as they may be at greater risk of missing out on appropriate eye care.

## 1. Introduction

Vision plays an important role in children’s development, emotional and social well-being, (Fazzi et al., 2010; Sonksen and Dale, 2002) and education (Bruce, Kelly, et al., 2018; Mathers et al., 2010). Visual problems are common in young children in the UK with an estimated 1–2% affected by amblyopia, 3–5% affected by strabismus and 20% affected by significant refractive error (Rahi, 2017). Furthermore, children with additional support needs (ASN) have a higher risk of visual problems than typically developing children (Das et al., 2010). Screening children at the age of 4–5 years allows time to correct any eyesight problems arising from the aforementioned conditions before the child starts school (Hall, 2001; Lowth, 2013; UK National Screening Committee, 2023). According to Bruce et al. (Bruce, Kelly, et al., 2018) successful spectacle wear can significantly improve visual outcomes in children taking part in a vision screening programme. The Scottish Government introduced the Preschool Orthoptic Vision Screening (POVS) program in 2012. The uptake of the initial screening has been consistently high (85.5%) according to national data from 2013–2016 across Scotland. Onward referral for a more detailed eye examination was offered to 17.9%. Eighty-nine percent of these children had a significant refractive error (Pentland and Patel, 2020). Depending on the local arrangements, children who screen positive during the POVS are referred for further assessment and treatment of their condition. In Edinburgh, a proportion of children are assessed in the Orthoptic/Optometry Joint clinic at the hospital eye service (HES). A high uptake of the initial screening ensures that eye problems are identified in the majority of children. However, it appears that a number of children do not attend for their scheduled HES appointment and are at risk of not receiving appropriate interventions to treat their condition. This concern has been flagged up by Bruce and Outhwaite (Bruce and Outhwaite, 2012) who evaluated attendance rates in Bradford. Low attendance rates for follow-up appointments are a concern in terms of patient safety, especially as the group of children needing a follow-up in the HES are likely to require spectacle correction. A qualitative study in Bradford (Bruce, Sanders, et al., 2018) showed that parental perceptions about the severity of visual issues in their children played an important role in attending eye appointments and adhering to spectacle wear. Other considerations included social influences, scheduling issues and information-sharing. The current audit presents quantitative data regarding the reasons for attendence and non-attendance patterns in children with and without ASN.

## 2. Description of POVS in Edinburgh

Orthoptist-led POVS is offered to all preschool children (aged 4–5) in the City of Edinburgh. Parents receive a letter through their nurseries to inform them about the POVS visit and have the opportunity to opt-out. Children who do not attend nursery or are absent from nursery on the day of the POVS are invited to a mop-up POVS visit at locality clinics across the city. If a child does not attend this appointment, their caregivers receive a letter informing them to attend a community optometrist if they have any concerns. Children who missed their POVS will have their eye test carried out by the school nursing team in Primary 1. The aim is that all children attending school have had their vision screening before their sixth birthday.

### Screening tests

During the POVS, the following tests are carried out:

– Visual acuity (VA) with or without glasses (Crowded LogMAR, Crowded Kays Pictures)
– Cover test
– Ocular movements
– Convergence
– Prism reflex (15/20 Dioptre)
– Frisby stereo acuity

### Outcome and planned follow-up

Children pass the POVS if they achieve a VA of 0.200 or below on Crowded LogMAR in one or both eyes, or below 0.100 in one or both eyes on Crowded Kay pictures with an intraocular difference of less than 3 optotypes. Parents of children who do not pass the criteria at the POVS receive a letter for a follow-up appointment within an appropriate clinic. After failing the POVS, ocular motility issues are subsequently assessed by orthoptists. Nerve palsies, neurological conditions and severely reduced vision are assessed by ophthalmologists. Children with mildly reduced vision are referred to the community optometrist. Children with moderately reduced vision, incomplete tests or complex needs are assessed in the Orthoptic/Optometry Joint clinic. Detailed referral criteria can be found in Appendix I. The current study concerns the Orthoptic/Optometry Joint clinic at the HES in Edinburgh. In this clinic, the vision tests from the POVS are repeated by the orthoptist. The orthoptist in the HES has a wider range of tests available, such as Cardiff Cards and the Lang stereo test, both of which are particularly suitable for children who do not respond to the tests used in the POVS. The HES assessment also includes a more detailed assessment of obstetric, medical, ocular and family history. The optometrist carries out a cycloplegic refraction and fundus examination. If needed, children receive a spectacle prescription and/or instructions for further management, such as occlusion therapy.

## 3. Aim of the study

This study evaluates the outcomes of children attending the Orthoptic/Optometry Joint clinic after failing the POVS in terms of refractive errors and vision improvement after spectacle correction. The attendance rate, barriers to attendance and inequality between children with ASN and without ASN are evaluated and ideas from caregivers for improving access to the HES are presented.

## 4. Methods

For this study, the authors had access to the annual national database for POVS for the 2021–2022 and 2022–2023 cohorts, the first six weeks of the 2023–2024 cohort in Edinburgh City and to the local hospital electronic records system (TRAK). Data was anonymised and transferred to an Excel spreadsheet.

### Data collection from TRAK and POVS database for 2021–2022, 2022–2023 and 2023–2024 cohorts

Data from all cohorts were used to identify the attendance rates for children attending the Orthoptic/Optometry Joint clinic after failing the POVS tests. The children were split into three groups depending on their attendance status: First-time attendees, attendees after one missed appointment and children who missed two or more appointments. For each group, their vision at the POVS visit and the presence of ASN were compared. For the groups of attendees, data about refractive errors was collected. Appendix II provides an overview of the criteria for refractive errors used for this study. Additionally, data from the 2021–2022 cohort was used to evaluate visual acuity after spectacle correction. These data were not available for the other cohorts at the time of data collection.

### Survey

Caregivers of children who failed to attend the Orthoptic/Optometry Joint clinic in the 2022–2023 cohort and the first six weeks of the 2023–2024 cohort were invited to participate in a telephone survey. In this survey they were asked about barriers to engaging with the HES and their views on improving their children’s access to the HES. The caregivers were first asked what the single main reason was for them not attending the clinic. Subsequently, caregivers could select multiple other barriers preventing them from attending. The questionnaire went on to establish whether caregivers were currently concerned about their child’s vision, whether the child had any subsequent ophthalmic examination since the POVS and had gone on to receive glasses and, if so, whether this was from the HES or the community. Finally, the caregivers were asked their opinion on potential improvements such as the quality of the referral letter, the location of the vision test and other improvements. The survey questions are listed in Appendix III. The purpose of the phone call was explained to the caregiver and the survey questions were only asked after the caregiver agreed to take part. After the telephone survey the anonymised data was transferred to an Excel spreadsheet. Consent was obtained for carers’ responses to be included for publication.

Ethical approval was obtained from the local health board (NHS Lothian) quality improvement and audit committee.

## 5. Results

### Participants

During the school year of September 2021–August 2022, a total of 888 children attended the POVS. Twenty percent of these children (n = 177) were subsequently referred to the Orthoptic/Optometry Joint clinic. Five children moved out of the catchment area before their appointment in the Orthoptic/Optometry Joint clinic and were subsequently excluded from the study. For another two patients, insufficient data were available, and they were also excluded. A total of 170 children of the 2021–2022 cohort were included in this study.

From September 2022–August 2023 a total of 701 children attended the POVS. Twenty percent of these children (n = 143) were subsequently referred to the Orthoptic/Optometry Joint clinic. Four children moved out of the catchment area or were not given an appointment in the Orthoptic/Optometry Joint clinic for other reasons and were subsequently excluded from the study. A total of 139 children from the 2022–2023 cohort were included in this study.

For the 2023–2024 cohort, data were available for the first six weeks, from September 2023–mid-August 2023. A total of 48 children were referred to the Orthoptic/Optometry Joint clinic after failing their POVS. Three children moved out of the catchment area or were not given an appointment in the Orthoptic/Optometry Joint clinic for other reasons and were subsequently excluded from the study. A total of 45 children from the 2023–2024 cohort were included in this study.

**Figure 1:**
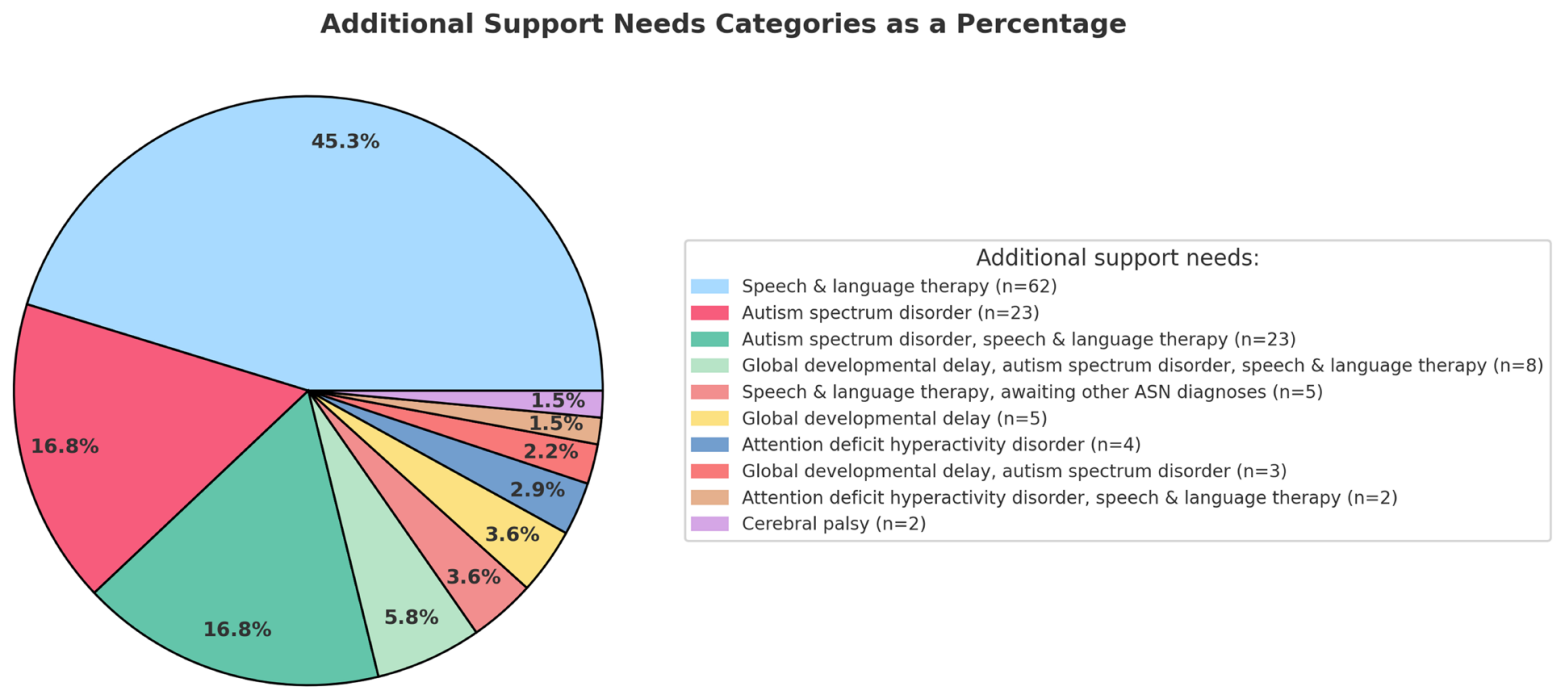
Additional support needs categories as a percentage for the combined cohorts.

Of the 354 children from the three cohorts, 56% (n = 199) were male and 44% (n = 155) were female. The average age of the children at the screening visit was 4.5 years. Two-fifths (n = 137) of the children had ASN. Figure 1 shows the distribution of ASN categories. Children with ASN were 1.8× more likely to miss two appointments compared to children without ASN. Visual acuity (VA) assessment was incomplete in 27% (n = 94) of all children in our sample at the screening visit. Eighty-eight percent of these children had ASN. Of the children with incomplete tests, 45% (n = 42) attended their first appointment, 13% (n = 12) missed one appointment and 43% (n = 40) missed two appointments in the Optometry/Orthoptic Joint clinic. Children with completed VA measurements (n = 259, 73%) at the POVS had an average of 0.25 ± 0.17 LogMAR in their better eye and 0.44 ± 0.44 LogMAR in their worse eye. On average, the VA in the better eye was 0.02 LogMAR worse in children with ASN (0.27 ± 0.19) compared to children without ASN (0.25 ± 0.16). The VA in the worse eye was 0.10 LogMAR better in children with ASN (0.37 ± 0.21) compared to children without ASN (0.47 ± 0.48). The independent-samples t-test (P < 0.05) showed that the difference in VA between children with and without ASN was not significant in either eye.

Table 1 provides an overview of participants’ characteristics.

**Table 1:** Attendance rates and characteristics of children invited to Joint Orthoptic/Optometry clinic after POVS.


	COHORT	SCENARIOS						COHORT TOTALS	

		ATTENDED		DNA ×1		DNA ×2			

**Attendance rate**	2021–2022	n = 101	59%	n = 20	12%	n = 49	29%	n = 170	100%

	2022–2023	n = 90	65%	n = 14	10%	n = 35	25%	n = 139	100%

	2023–2024	n = 25	56%	n = 7	16%	n = 13	29%	n = 45	100%

	**Cohorts combined**	**n = 216**	**61%**	**n = 41**	**12%**	**n = 97**	**27%**	**n = 354**	**100%**

**Number of children with incomplete VA measurement at POVS visit in each scenario**	2021–2022	n = 15	15%	n = 5	25%	n = 15	31%	n = 35	21%

	2022–2023	n = 20	22%	n = 3	21%	n = 17	49%	n = 40	29%

	2023–2024	n = 8	32%	n = 3	43%	n = 8	62%	n = 19	42%

	**Cohorts combined**	**n = 43**	**20%**	**n = 11**	**27%**	**n = 40**	**41%**	**n = 94**	**27%**

**Number of children with additional support needs in each scenario**	2021–2022	n = 35	34%	n = 7	35%	n = 25	51%	n = 67	39%

	2022–2023	n = 31	34%	n = 5	36%	n = 20	57%	n = 56	60%

	2023–2024	n = 10	40%	n = 4	57%	n = 8	62%	n = 22	49%

	**Cohorts combined**	**n = 76**	**35%**	**n = 16**	**39%**	**n = 53**	**55%**	**n = 145**	**41%**


Attended: Children who arrived for their first appointment. 1× DNA: Children who missed their first appointment and attended for their second appointment. 2× DNA: Children who missed the first and second appointment.

Data about spectacle prescribing was available for 62% (n = 221) of children. Over two-thirds (n = 151) of these children required a spectacle prescription. Refractive errors included: 12% emmetropia (n = 27), 35% hypermetropia (n = 78), 19% hypermetropia with astigmatism (n = 43), 9% myopia (n = 19), 16% myopia with astigmatism (n = 35) and 7% mixed astigmatism (n = 16). Anisometropia was diagnosed in 32% of children (n = 70).

VA was measured before and after spectacle wear in children who attended the Orthoptic/Optometry Joint clinic. A total of sixty-three children completed VA measurements before and after spectacle wear at the time of data collection. In these children, the paired-samples t-test (p < 0.05) demonstrated a significant improvement in VA after spectacle correction. An average VA improvement of 0.07 logMAR was measured in the better eye: from 0.27 ± 0.16 LogMAR before spectacle correction to 0.20 ± 0.14 LogMAR after spectacle correction. In the worse eye, the average VA improvement was 0.16 LogMAR: from 0.42 ± 0.18 LogMAR before spectacle correction to 0.26 ± 0.16 after spectacle correction.

### Surveys

**Figure 2:**
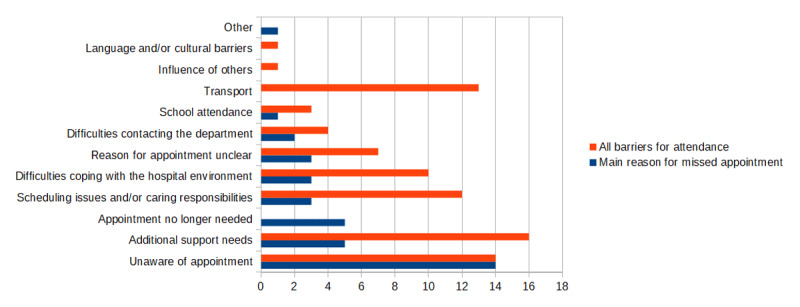
Main reason for missed appointment and barriers to attendance.

Sixty-nine caregivers were identified as suitable participants for the phone survey. Phone numbers were not available for five people and 27 potential participants did not answer the phone. Thirty-seven agreed to be interviewed and successfully answered all the questions in the survey. Four of the participants had one missed appointment and 23 had two missed appointments in the HES. Figure 2 outlines the main reason for missed appointments as well as all the barriers for attendance as reported by the carers. The single response grouped under the ‘other’ category was due to the family being away on holiday during the time of the appointment. Precise reasons for why the appointment was thought to be no longer needed included two instances of the HES waiting time being perceived by the caregiver to be too long (the children were instead taken to a community optometrist); two instances of families moving out of Scotland; and one instance of the caregivers being unconcerned with their child’s vision, assuming the referral was only due to their child being unable to complete the POVS screening and hence unnecessary. Along with the 11 barriers displayed, carers were also given the option of indicating the style of the referral letter and the cost of glasses as barriers, however, none did so.

Twenty-three per cent (n = 7) of caregivers were actively concerned about their child’s vision at the time of the survey. Forty-three per cent (n = 16) of children had received an ophthalmic examination since the time of the POVS. Out of these, nine received glasses—seven from the community and two from the HES. Of the remaining seven, five were examined in the community and two in the HES.

**Figure 3:**
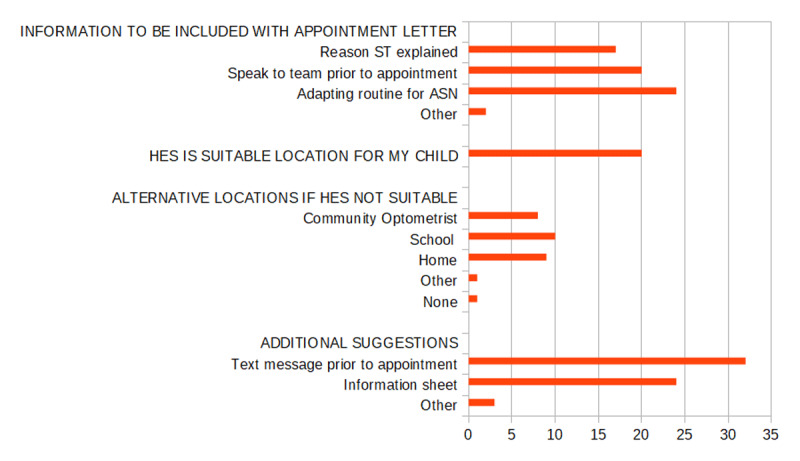
Suggestions from caregivers regarding improving eye care service access.

Caregivers placed importance on the content of the referral letter and suggested including a clear explanation of the reasons and benefits of their child’s sight test, a contact number for the clinic and information on how the assessment could be adapted to better suit children with ASN. Figure 3 shows the responses from the participants in more detail. The responses grouped under the ‘other’ category were options for alternatives to the HES clinic appointment; an explanation of the appointment and the examinations performed; and an approximation of the duration time of the appointment. Whilst 54% (n = 20) indicated that the HES was a suitable location for their child’s sight test, 46% (n = 17) of caregivers considered the HES an unsuitable venue for the clinic. Figure 4 lists suggested alternative locations. The responses grouped under the ‘other’ category were local health clinics and the Royal Hospital for Children & Young People. Other potential improvements are also listed in Figure 3.

Lastly, caregivers were asked for any final general comments. Three of these concerned the POVS, one caregiver felt the POVS was rushed; the second would have preferred to have been made aware in advance that screening would take place and felt this may have enabled them to prepare their child better, thus avoid failing the POVS; the third felt that their child was too young for the POVS. A further caregiver expressed their child’s discomfort relating to eye drops. Finally, a caregiver identified poor concentration as the reason for the child failing the POVS.

## 6. Discussion

### Audit

In keeping with previous studies (Hall, 2001; Lowth, 2013; Pentland & Patel, 2020; Solebo & Rahi, Jugnoo S., 2019; UK National Screening Committee, 2023; Williams, 2009), our audit shows the benefit of vision screening in children aged 4–5 years. In our study at least two thirds of the children attending the Orthoptic/Optometry Joint clinic required spectacle correction, leading to improved VA, especially in the worse eye. Poor VA in one eye is often not picked up by parents, which highlights the importance of the POVS program. The difference in VA between children with and without ASN was not statistically different although a clinically significant difference existed. A future study with a larger cohort within the current setting or a multi-centre study could explore this further. A wider range of tests at the POVS to increase the amount of complete tests would further improve a true reflection of VA at screening as there was a particularly high rate of incomplete tests in the ASN group. A lower amount of incomplete screening tests would also reduce the amount of HES appointments required.

The consequences of missed HES appointments are two-fold: it presents a financial burden to the HES and it presents a risk for patients whose visual issues are not addressed. To address the financial burden, the HES in Edinburgh has changed the appointment policy for children failing the POVS since the time of the audit: children with complex needs or incomplete tests are now offered a single appointment in the Orthoptic clinic rather than a double appointment slot in the Orthoptic/Optometry Joint clinic. Furthermore, for all children who missed a HES appointment, carers now receive a discharge letter with an option to request another appointment, rather than a second appointment. In terms of risks for patients, the HES needs to continue to ensure that patients receive the care they require at the right time. This study showed that children with ASN had a higher rate of incomplete screening tests and missed HES appointments, potentially leading to missing out on appropriate eye care. This concern has been raised by Donaldson et al. (2019) who demonstrated a high prevalence of previously undetected vision problems in children attending special schools, despite the presence of a school-entry vision screening program.

### Survey

The most common reason given for not attending the Optometry/Orthoptics Joint clinic was that caregivers were unaware of the HES appointment. Although the parents of the 2021–2022 cohort were not invited to participate in the survey to minimise the time lag between the appointment being offered and the time of the survey, it is still possible that the time lag between appointment and interview resulted in the participants having forgotten about the HES appointment at the time of the survey. However, it is also possible that they did not receive the appointment letter or did not understand the purpose of the letter. The appointment letter being sent to parents in Edinburgh is a standard hospital letter containing information which is relevant to adult clinics as well as additional information specific to paediatric clinics. Cassetti et al. (Cassetti et al., 2019) interviewed eye care professionals about ideas to improve attendance rates in similar clinics and recommended personalised communication, better healthcare education and improved healthcare pathways. The caregivers in our study likewise appreciated information about their child’s eye health in an accessible format, along with information about the clinic appointment and how the eye test could be adapted for children with ASN. They also appreciated having an option to speak with a member of staff about special arrangements and rescheduling. An information leaflet in accessible format for children and their caregivers can be sent along with their appointment letter.

Our study showed that parents appreciated the idea of receiving a reminder for their appointment in the form of a text message. This method is an easy and effective way to reduce the risk of patients not attending in adult services (Car et al., 2008; Gurol-Urganci et al., 2013). However, this method is not permitted for paediatric services due to local policy. Reminder letters and phone calls to explain the reason for referral to caregivers of children who failed the POVS are now being considered.

Besides the issue of being aware of an upcoming appointment, this study has shown that ASN plays a key role in preventing patients and their caregivers from attending. One concern is that the hospital environment is perceived to be a barrier to attendance. Parents are worried that their child may not cope with a standard test and require alternative arrangements in terms of appointment time and adaptations to the usual routine. Parents would benefit from understanding that the eye care professionals in the HES are experienced in assessing and managing children with ASN. Another concern is the logistical challenge of managing their caring responsibilities and organising transport. Our survey shows that a significant proportion of caregivers prefer an alternative location, such as community optometrists, school or home. Careful consideration is required when other locations are considered as one needs to ensure that the person carrying out the test has sufficient experience in assessing children with ASN.

None of the caregivers surveyed indicated the cost of glasses as a barrier to attending the clinic. This makes sense considering the free and universally accessible provision of eye examination and NHS optical vouchers for glasses in Scotland (Jonuscheit et al., 2019; Kearney et al., 2022; Legge et al., 2018).

### Bias and limitations

The survey questions were presented with model answers for ease of data collection and processing, which leads to a level of bias. To minimise this effect, at each point, opportunity was given for the caregivers to express their opinions freely under the ‘other’ category. The questionnaire was carried out by one of the investigators, rather than anonymously. Whilst this method had practical advantages and was thought to trigger the best response rate, this method may have caused some bias. As mentioned before, there was inevitably a time lag between the time of the HES appointment being offered and the time the survey was taken, leading to more potential for error in recollecting the main reason for not attending.

## 7. Conclusion

This study has shown that, whilst the initial uptake of POVS is high, there is room for improvement in terms of reducing the number of incomplete tests at screening and the rate of missed appointments in the HES. Particular attention should be given to making the service more accessible for children with ASN as they are more at risk of missing out on appropriate eye care.

## Additional Files

The additional files for this article can be found as follows:

10.22599/bioj.451.s1 Appendix I. Pre- School Orthoptic Screening Referral Criteria.

10.22599/bioj.451.s2 Appendix II. Criteria for refractive errors, assuming a minus cylinder notation.

10.22599/bioj.451.s3 Appendix III. Survey questions for care-givers.
