# Supplementary material for: Children with Additional Support Needs Risk Missing Out on Effective Vision Screening: Audit and Survey Considering Attendance Rates and Parent Reported Barriers to Service Access, including Recommendations for Improvement
Source: Br Ir Orthopt J. 2025 Apr 3;21(1):43–50. doi: 10.22599/bioj.451 (PMC11987887; doi:10.22599/bioj.451)
Supplement: Appendix I. — Pre- School Orthoptic Screening Referral Criteria. [file bioj-21-1-451-s1.pdf]

# APPENDIX I

Pre- School Orthoptic Screening Referral Criteria (last updated January 2018)

NB: Referral criteria have been updated since the period of the audit. Children with ASN are now referred to the Hospital Orthoptic clinic

| **Hospital Ophthalmologist**<br><br>**(PAEDS CLINIC)**<br>**+ OD** | **Hospital Joint Orthoptic/Optometry Clinic** | **Community Optometrist**<br>**+**<br>**Orthoptist** | **Community Optometrist Only** |
|---|---|---|---|
| **Cr.LogMAR**:<br>< 0.8 in one or both eyes<br><br>**Cr.Kays**:<br>< 0.7 in one or both eyes | **Cr.LogMAR**:<br>0.775 - 0.5 in one or both eyes<br><br>**Cr.Kays**:<br>0.675 - 0.4 in one or both eyes | **Cr.LogMAR**:<br>0.475 - 0.325 in one or both eyes<br><br>**Cr.Kays**:<br>0.225 - 0.375 in one or both eyes | **Cr.LogMAR**:<br>0.225 - 0.300 one or both eyes<br><br>**Cr.Kays**:<br>0.125 - 0.200 in one or both eyes |
| **Severe** Additional Needs or neurological condition(e.g hydrocephalus) * | Additional Needs** | Poor compliance (**not** due to ASN) | Intraocular difference of 3 optotypes |
| Lid and Pupil Abnormalities (eg.ptosis, heterochromia, coloboma,pupillary margin lesion) | Esotropia:<br>Constant or Intermittent<br><br>Exotropia:<br>Intermittent with reduced VA | Pass level VA achieved with **significant** esophoria present with fail of 1 or 2 BV tests | Pass level VA achieved with pass in both BV tests(prism reflex/frisby) but **slight** esophoria (less than 10 dioptres eso) |
| Ocular Motility defect (eg. Browns, Duanes, SO weakness)<br>(not physiological V tendency) | | Pass level VA but reduced near point of convergence | |
| Nystagmus | | | |
| Constant Exotropia | | | |
| Severe Inflammation | | | |

| | | |
|---|---|---|
| PASS Criteria | **Cr.LogMAR**:<br>> 0.200 one or both eyes<br><br>**Cr.Kays**:<br>> 0.100 one or both eyes<br>Intraocular difference of less than 3 optotypes | NAD<br>Exophoria, Esophoria (non-significant) |

*and**:  Use professional judgement
