# Supplementary material for: Children with Additional Support Needs Risk Missing Out on Effective Vision Screening: Audit and Survey Considering Attendance Rates and Parent Reported Barriers to Service Access, including Recommendations for Improvement
Source: Br Ir Orthopt J. 2025 Apr 3;21(1):43–50. doi: 10.22599/bioj.451 (PMC11987887; doi:10.22599/bioj.451)
Supplement: Appendix II. — Criteria for refractive errors, assuming a minus cylinder notation. [file bioj-21-1-451-s2.pdf]

APPENDIX II

Criteria for refractive errors, assuming a minus cylinder notation adapted from Galvis (Galvis, 2021)

	**Spherical equivalent (absolute value)**	**Cylinder value**	**Absolute Sphere value (assuming minus cylinder notation)**
**Emmetropia**	-0.25D to +0.25D	0.75D or less	
**Myopia**	-0.50D or more minus		Zero or more minus (in addition to spherical equivalent of -0.50 or more minus)
**Myopia + Astigmatism***	-0.50D or more minus	1D or more	Zero or more minus (in addition to spherical equivalent of -0.50 or more minus)
**Hyperopia**	+0.50 or more plus	Cylinder value equal or less than value of sphere	
**Hyperopia + Astigmatism***	+0.50 or more plus	Cylinder value equal or less than value of sphere<br><br>AND<br><br>Cylinder value -1D or more minus	
**Mixed Astigmatism**		Cylinder value greater than value of sphere<br><br>AND<br><br>Cylinder value -1D or more minus	Zero or hyperopic
**Anisometropia****	1D or more difference between eyes	1D or more difference between eyes	

*Galvis et al. combined hyperopia and hyperopia+astigmatism into one group and also myopia and myopia+astigmatism
**Galvis et al. did not include anisometropia

References

Galvis, V. (2021). Definition of refractive errors for research studies: Spherical equivalent could not be enough. *Journal of Optometry*, *14*, 224–225.
