# Supplementary material for: Children with Additional Support Needs Risk Missing Out on Effective Vision Screening: Audit and Survey Considering Attendance Rates and Parent Reported Barriers to Service Access, including Recommendations for Improvement
Source: Br Ir Orthopt J. 2025 Apr 3;21(1):43–50. doi: 10.22599/bioj.451 (PMC11987887; doi:10.22599/bioj.451)
Supplement: Appendix III. — Survey questions for care-givers. [file bioj-21-1-451-s3.pdf]

APPENDIX III

Survey questions for care-givers

Are you concerned about your child's vision? □ Yes □ No

What is your main reason for missing the appointment? Tick boxes and write additional information if applicable

Communication with HES	□ Did not realise I had an appointment/ did not receive appt letter
	□ Did not understand reason for appointment
	□ Difficulties contacting department to cancel or reschedule appointment
	□ Letter was off-putting
Priorities and logistics	□ Unable to get transport/ transport too expensive/ parking too expensive
	□ Did not want to miss school
	□ Scheduling issues (eg work or caring responsibilities)
Personal barriers	□ Cultural/language barriers
	□ Worried about costs for glasses
	□ Additional support needs
	□ Hospital environment causing trauma/anxiety
	□ Influence of other people's experiences
	□ Appointment no longer needed (state reason why)
Other	□ Describe

Have you ever experienced any of the following barriers for attending HES appointments for your child?

Communication with HES	□ Did not realise they had an appointment
	□ Reason for appointment unclear
	□ Difficulties contacting department to cancel or reschedule appointments
	□ Letter style
Priorities and logistics	□ Difficult to get transport/ transport too expensive/ parking too expensive
	□ Not wanting to miss school
	□ Scheduling issues (related to work or caring responsibilities)
Personal barriers	□ Cultural/language barriers
	□ Worried about costs for glasses
	□ Additional support needs
	□ Hospital environment causing trauma/anxiety
	□ Influence of other people's experiences

Other □ Describe

Has your child had an eye test since the POVS? Where? Did they need glasses?

□ No

□ Yes, HES, No glasses

□ Yes, HES, glasses

□ Yes, GOS/Optician, no glasses

□ Yes, GOS/optician, glasses

What information would you find helpful in the invitation letter?

□ Benefits/need for sight test for your child explained

□ Option of speaking to the eye team before the appointment

□ Options for children who do not cope with drops/hospital environment/ How routine can be adapted

□ Other

What can the HES do to make it more accessible for children in your situation?

□ Text message prior to appt

□ Letter/info sheet explaining purpose of appt

□ Other

Would you prefer the sight test in a different location

□ HES is fine

□ Community optometrist

□ School

□ Home

□ Other

Have you got any other suggestions for making eye care more accessible within or outside the HES?
